# Essential neonatal care utilization and associated factors among mothers in public health facilities of Aksum Town, North Ethiopia, 2016

**DOI:** 10.1371/journal.pone.0175902

**Published:** 2017-04-19

**Authors:** Megbey Berhe, Araya Abraha Medhaniye, Gizienesh Kahsay, Ermyas Birhane, Mebrahtu Abay

**Affiliations:** 1 Department of Public Health, Aksum University, Aksum, Ethiopia; 2 School of Public Health, Mekelle University, Mekelle, Ethiopia; Centre Hospitalier Universitaire Vaudois, FRANCE

## Abstract

**Background:**

Globally, neonatal death accounts about 44% of child death in 2013. Ethiopia is one of the ten countries with the highest number of neonatal death. Worldwide, more than 43% of deaths among under five year children is contributed by neonates. Half of the neonatal death occur in the first day of life. Recommendations about newborn care practices may conflict with local beliefs and practices. So, it is important to understand the existing newborn care practice and factors affecting it in order to take interventions so as to decrease neonatal death.

**Objective:**

To assess magnitude of essential neonatal care utilization and associated factors among women visiting public health facilities in Aksum Town, Tigray, Northern Ethiopia, 2015.

**Methods:**

Facility based cross sectional study was conducted from December 30, 2015 to January 31, 2016.The sampled population are 423 women who gave live births within the last 6 months prior to data collection. Systematic random sampling technique was employed. Data were entered, coded and cleaned using Epi info version 7, and SPSS Version 21 software was used for analysis. Both bivariable and multivariable logistic regression models were used to determine factors associated with essential neonatal care utilization. Variables with P-value <0.2 in the bivariable logistic regression model were included in to multivariable logistic regression model, and finally variables with P-value <0.05 were considered as independent factors. Odds ratio was used to measure strength of association at 95% confidence level.

**Result:**

A total of 423 mothers included in the study. Prevalence of safe cord care, optimal breast feeding, thermal care and baby received Tetracycline eye ointment and vaccine at birth were 42.8%, 63.1%, 32.6% and 44.7% among the respondents respectively. Only **113(26.7%)** of the participants fulfilled essential new born care practice. Occupation, parity and counseling on essential new born care during delivery were significantly associated with utilization of essential new born care. Employed women (AOR = 7.08; 95% CI (2.21, 12.72), 2–3 number of deliveries (AOR = 1.84; 95% CI (1.04, 3.26) and received counseling about essential new born car during delivery (AOR = 3.36; 95% CI (1.86, 6.08) were more likely to practice essential neonatal care practice than their counterparts.

**Conclusion and recommendation:**

Around three-fourth of mothers were not practicing Essential Newborn Care (ENC). Occupation, parity and essential new born care counseling during delivery were significantly associated with utilization of ENC. Promotion of information at community level, women empowerment and staff training is recommended.

## Background

Globally, neonatal death accounts about half of child death in 2013 and Sub-Saharan Africa (SSA) has the highest rates of neonatal mortality [1]. Asphyxia in the first day, preterm during the first week, and infections after the first week of life are the most common causes of death in the neonatal period [2]. Forty-eight percent and fifty two percent of neonatal death are due to lack of appropriate care given to mothers and neonates respectively, and most of those deaths could be prevented through low cost interventions like integrated antenatal care, quality care at birth and essential newborn care practice [3].

The world health organization (WHO) defined essential neonatal care as a comprehensive strategy designed to improve the health of newborns through interventions before conception, during pregnancy, at and soon after birth and in the postnatal period [4]. To save the life of neonates, the recommended WHO essential newborn care practice is a crucial intervention. ENC include clean cord care, thermal protection, early and exclusive breastfeeding, eye care, immunization at birth, care for the low birth weight newborn and management of newborns[5]. Universal coverage of these essential interventions would reduce neonatal death by an estimation of 71%[6].

Ethiopia is one of the country with the highest neonatal death in SSA. Annually, around 122,000 newborn death occur in the country[7]; this accounts for about half (37 per 1000) of all under-five children deaths. About 62% of infant death occur during the first month of life[8]. Birth order, frequency of antenatal care use, place of delivery, gestation age at birth, presence of premature rupture of membrane, complication during labor, twin delivery, low birth weight and neonatal care practice were identified as determinants of neonatal mortality[9]. Maternal age, timing of the first antenatal care visit and knowledge of the mothers on new-born danger signs are factors associated with good neonatal feeding practice [10]. Breastfeeding, new-born resuscitation, ‘kangaroo mother care’ for premature babies (prolonged skin-to-skin contact with the mother) and preventing and treating infections are the most effective interventions in saving life of new-borns [11].

To reduce neonatal death, Ethiopian government did many health interventions such as training midwifes, enhancing referral system, integrating health services, implementing packages of Health Extension Program (HEP) and routine immunization [12]. But neonatal death is still high, even one of the top ten countries in Africa. To improve survival of new-borns, essential new born care practice is a priority intervention method and can be applied through newborn resuscitation, early identifying and managing neonatal infections, addressing barriers to exclusive breast feeding, applying kangaroo mother care (KMC), appropriate umbilical cord care and improving quality of health care during delivery[13]. However, the care given to newborns is still low with inequitable coverage and high degree of variability in the utilization of essential new-born practices [6, 10, 14].

Studies on essential new born care practice are few in Ethiopia, especially in Tigray. Therefore, the aim of this study is to assess magnitude of newborn care practice and associated factors in the study area.

## Methods and materials

### Study design and setting

Health facility based cross-sectional study design was employed. The study was conducted in public health facilities of Aksum Town, Central Zone of Tigray, Northern Ethiopia. Aksum Town was the original capital of kingdom of Aksum which is around 1,024 Kilometers away from Addis Ababa, the capital city of Ethiopia. The Town has five Kebelles having a total of 13,810 households and 60, 676 population (3,0945 males and 29,731 females). It has one referral hospital, one zonal hospital, two health centers, one family guidance clinic, five private clinics, three private pharmacies, eight private drug shops and one rural drug vendor. There are 18 health extension workers assigned at Kebelle level. The number of reproductive age group women in the town during the data collection period are 13,956. Data collection for this study was conducted from December 30, 2015 to January 30, 2016. All women who gave live births within the last six months prior to the data collection and visited the public health facilities of Aksum Town were the source population.

### Sample size determination and sampling procedure

The sample size was calculated using a single population proportion formula with the assumptions of 95% confidence interval,5% of marginal error and p-value of 0.479 from a study done in Ethiopia [15]. Adding 10% non-response rate, the total sample size was 423.

In Aksum Town, there is one hospital, two health centers and one family guidance clinic which provide Maternal and Child Health (MCH) services. To determine the number of women to be interviewed from each health facility, data on client flow was considered and obtained from the health facilities, and the total sample size determined was distributed proportionally among each of the four health facilities. To select each woman from each health facility, systematic random sampling technique was employed.

### Variables and measurement

Essential neonatal care: a care includes all of the following things; safe cord care, thermal care, optimal breast feeding, TTC eye ointment and vaccination at birth.

Knowledge of mothers: mothers who score mean and above on the new born danger signs took as knowledgeable mothers.

Safe cord care: a cord care using clean instrument to cut umbilical cord and with no substance applied on it.

Optimal breast feeding: initiation of breast feeding with in the first one hour of delivery and no additional feeding given.

Thermal care: dry and wrapping of the new-borns, delay bathing of the newborns until 24 hours of age and skin to skin contact of the new-borns with their mothers.

### Data collectors and data collection procedure

A structured interview administered questionnaire, developed after reviewing different literatures, was used to interview women. First, the questionnaire was prepared in English and then translated in to the local language, Tigrigna and again back translated to English keep consistency of the data. Pretest was done on 5% of the total sample size at Adwa Town, a Town which is found 25 Kilometers away from the study area. Three nurse diploma data collectors and one BSc nurse supervisor were used for collection and supervision of the data. The supervisors were responsible for supporting the data collectors, checking filled out questionnaires daily for completeness and providing feedback for data collectors. The principal investigator also participated in the supervision of the data collection.

### Data quality assurance method

Three data collectors and one supervisor were trained for one day. They were trained on procedures of data collection techniques, approaching participants, ethical issues quality of data and advantage of collecting the actual data. The questionnaire was pretested on mothers who gave live births. Clarity, consistency, skipping pattern and order of the questions was done before the actual data collection period, and modification was done accordingly. Onsite supervision was carried out during the whole period of data collection in daily basis. At the end of each day, questionnaires were reviewed and cross checked for their completeness, accuracy and consistency by the principal investigator and corrective measures was under taken.

### Data processing and analyses procedures

Data were coded and entered in to a computer using epi-info version 7 and exported to SPSS version 21 software for analyses. Any logical and consistency error identified during data entry was corrected after revision of the original completed questionnaire. Descriptive statistics was employed using frequencies and percentages. Both bivariable and multivariable logistic regression models were used to determine factors associated with essential neonatal care utilization. Model of fitness was checked by Hosmer and Lemeshow test and its p-value was 0.807. To identify factors associated with essential new born care utilization, variables with p-value < 0.2 in the bivariable analyses were entered into multivariable logistic regression model and those with p-value <0.05 in the multivariable logistic regression model were considered as independent factors. Crude and adjusted Odds ratios were computed for each explanatory variable to determine the strength of association at 95% Confidence Interval (CI).

### Ethical considerations

The study protocol, including the consent papers, were approved by the Institutional Review Board of Aksum University, college of health sciences. After securing permission from the health facilities, written informed consent was obtained from each participant and participants were informed about their right to skip a specific question or completely withdraw from the interview at any time. They were ensured that their response will be kept confidentially.

## Result

### Socio demographic and economic characteristics

A total of 423 mothers were included in the study, with response rate 100%. The largest age distributions of the respondents were between the ages of 20 and 29(61.5%). Majority of the participants were married 396(93.6%), and Orthodox religion followers accounted for 388(91.7%) of the total participants. From the total, 285(67.4%) attended secondary school. Three hundred twenty-five (76.8%) of the respondents were housewives by occupation. Three hundred eighty-eight (91.7%) of mothers were living in urban setting. More than half of the study participants earn an income of above 500 Ethiopian birr per month (Table 1).

**Table 1:** Socio demographic characteristics of the mothers in Aksum Town, Tigray, Ethiopia 2016.

Variables		Number	Percent
Age	15–19	28	6.6%
	20–29	260	61.5%
	30–39	130	30.7%
	40 and older	5	1.2%
Marital status	Single	20	4.7%
	Married	396	93.6%
	Others	7	1.7%
Educational status	Unable to read and write	34	8.0%
	able to read and write	18	4.3%
	Elementary	126	29.8%
	Secondary	185	43.7%
	college and above	60	14.2%
Occupation	house wife	325	76.8%
	Student	10	2.4%
	Employed	69	16.3%
	Merchant	19	4.5%
Religion	Orthodox	388	91.7%
	Muslim	35	8.3%
Residence	Rural	35	8.3%
	Urban	388	91.7%
House hold income	<650	25	5.9%
	651–1400	95	22.5%
	1401–2350	137	32.4%
	2351–3550	85	20.1%
	3551–5000	63	14.9%
	5001and above	18	4.3%

### Maternal health services related characteristics of respondents

From the total respondents, 412 (97.3%) gave birth at health facility, 410 (93.7%) gave birth at term, 254 (60%) were not visited by Health Extension Workers (HEW) within the first week of delivery. Almost all, 419 (99%), respondents can reach within one hour to their nearby health facility. Majority of the mothers, 267(63.1%) were multi parous, 420 (99.3%) have attended antenatal care (ANC) in their last pregnancy, 393 (92.9%) counseled on nutrition during ANC follow up. More than half of the mothers, 226 (53.4%), started ANC follow up earlier than three months, 235 (55.6%) knew about newborn danger signs. Majority of the respondents, 380 (89.8%), were assisted by a nurse or a midwife during their ANC follow up, 31 (7.3%) followed by doctor, 11 (2.6%) followed by a relative and only one respondent followed by HEW in their last delivery. From the total respondents, 396 (93.6%) were weighed their newborn at birth, of these 15 (3.5%) were low birth weight and only four of the low birth weight newborns received skin to skin contact with their mothers (Table 2).

**Table 2:** Maternal health service of respondents, in Aksum Town, Tigray, Ethiopia, 2016.

Variables		Number	Percent
Gestational age at birth	less than 37week	13	3.1%
	37week or more	409	96.9%
HEW visit in the first week	No	254	60.0%
	Yes	169	40.0%
Distance from health facility	<60 min	419	99.1%
	> = 60min	4	0.9%
Number of pregnancy	1	152	36.1%
	2–3	197	46.8%
	4 and above	72	17.1%
Number of delivery	1	156	36.9%
	2–3	195	46.1%
	4 and above	72	17.0%
Number of ANC visit	One	0	0.0%
	Two	16	3.8%
	Three	93	22.2%
	Four	310	74.0%
GA at first ANC	3	226	53.8%
	3–6	187	44.5%
	7 and above	7	1.7%
Knowledge of danger signs	No	238	56.3%
	Yes	185	43.7%
Counsel during delivery	No	157	38.1%
	Yes	255	61.9%

### Knowledge of respondents on danger signs of the new born

One hundred eighty-five (43.7%) of the respondents have knowledge about newborn danger signs. Newborn danger signs mentioned by the mothers were fever, 340(80.4%) followed by poor suckling 257(60.8%). Majority of the neonates experienced poor suckling, 271(64.1), followed by difficulty of breathing 30(7.10%) and chest in drawing, 5(1.2%). From those who experienced danger signs, 287(67.8%), 43(10.2%), 5(1.2%) and 5(1.2%) get sick care from traditional healer, from governmental health facility, from private clinic and not received care at all, respectively.

### Prevalence of utilization of Essential Newborn Care

**Fig 1:**
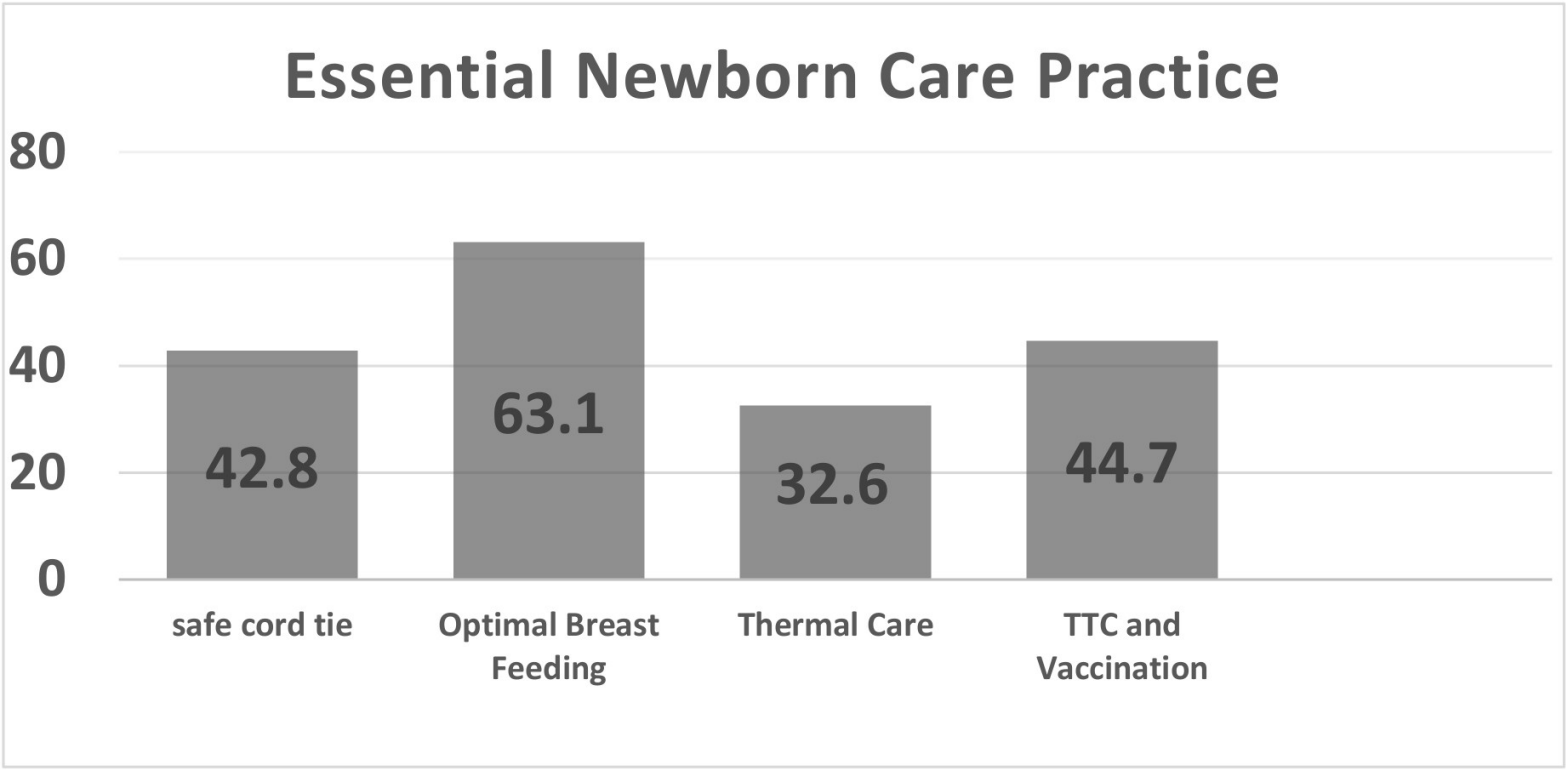
Four essential new born care practices in Aksum Town, Tigray, Northern Ethiopia.

From the total 423 mothers, 181(42.8%), 267 (63.1%), 138(32.6%) and 189 (44.7%) of the respondents practiced safe cord care, optimal breast feeding, thermal care with TTC eye ointment and vaccine at birth, respectively. But only 113 (26.7%) of participants fulfilled all the essential new born care practices (Fig 1).

### Factors associated with utilization of essential new born care

On bivariable analyses, educational status, occupation, marital status, residence, house hold income, gravidity, parity, frequency of ANC visit, gestational age of pregnancy at first ANC, place of delivery, essential new born care counseling during delivery, ever heard about essential new born care and visiting by health extension workers in the first week were associated with essential new born care utilization.

On multivariable Logistic regression analyses, Occupation, parity and essential new born care counseling during delivery were the only significantly associated factors with utilization of essential new born care. Employed women were 7.08 times more likely to practice essential new born care practice than housewives with AOR = 7.08; 95% CI (2.21, 2.72). Women who have 2–3 number of deliveries were 1.84 times more likely to practice essential new born care practice than women who gave birth once with AOR = 1.84; 95% CI (1.04, 3.26). Women who received counseling during delivery were 3.36 times higher to practice essential new born care as compared to women who did not receive counseling with AOR = 3.36; 95% CI (1.86, 6.08) (Table 3).

**Table 3:** Factors associated with ENC utilization by multiple logistic regression analysis, Aksum Town, Tigray Ethiopia 2016.

Variable	ENC		COR (95%CI)	AOR (95% CI)
	No	Yes		
Counseling during delivery				
No	133	24	1	1
Yes	168	89	2.94(1.77–4.86)	3.36(1.86–6.08)*
Educational status				
Unable to read and write	28	6	1	1
Able to read and write	13	5	2.40(.78–7.42)	1.49(.29–7.70)
Elementary	96	30	1.83(.57–5.93)	2.47(.47–12.94)
Secondary	139	46	2.10(.63–6.98)	1.69(.44–6.56)
College and above	34	26	4.20(1.02–7.32)	1.93(.57–6.60)
Residence				
Rural	31	4	1	1
Urban	279	109	3.03(1.04–8.78)	.43(.13–1.43)
Number of deliveries				
1	122	34	1	1
2–3	135	60	1.59(0.98–2.59)	1.84 (1.04–3.26)*
4 and above	53	19	1.28(0.67–2.46)	1.70(.77–3.75)
Marital status				
Single	18	2	1	1
Married	286	110	3.46(.79–15.17)	5.00(.84–29.79)
Others	6	1	1.50(.12–19.64)	2.02(.12–34.61)
Heard about ENC				
No	59	8	1	1
Yes	251	105	3.09(1.43–6.68)	1.19(.49–2.89)
HEW visit in the first week				
No	194	60	1	1
Yes	116	53	1.48(.96–2.28)	1.16(.71–1.90)
Occupation				
House wife	253	72	1	
Student	10	0		1
Employed	35	34	3.41(1.99–5.86)	7.08(2.21–12.72)*
Merchant	12	7	2.05(0.78–5.40)	2.25 (.74–6.83)
Income				
<650	21	4	1	1
651–1400	77	18	1.23(.37–4.02)	.48(.13–1.82)
1401–2350	94	43	2.40(.78–7.42)	.90(.23–2.05)
2351–3550	63	22	1.83(.57–5.93)	.53(.14–2.05)
3551–5000	45	18	2.10(.63–6.98)	.51(.12–2.10)
5001and above	10	8	4.20(1.02–17.3)	.68(.12–3.66)

COR: Crude Odds Ratio, AOR: Adjusted Odds Ratio, CI: Confidence interval.

*Significant at p-value <0.05.

## Discussion

The finding of this study showed that the proportion of newborn babies who received essential newborn care practices were 26.7%. Occupation, parity and essential new born care counseling during delivery were factors significantly associated with essential new born care practice.

The prevalence of essential new bore care practice in this study is consistent with another study conducted in east Gojam Zone, Ethiopia (23.1%) [16]; but it is lower than other studies conducted in Northwest Ethiopia (40.6%) and Addis Ababa (38.8%) [17, 18]. The possible explanation for the difference could be the socio-economic difference between the study areas, and Addis Ababa is more urbanized as compared to this study area.

Employed participants were 7 times more likely to utilize essential new born care than housewives. This can be due to the fact that employed mothers are expected to be more educated, and educated mothers can have better understanding about essential new born care practice than their counter parts.

Parity was also found to be significantly associated with essential new born care practice. Mothers who gave birth 2 or 3 were 1.8 times more likely to utilize essential new born care than those who gave birth once. A similar study conducted in six low and middle income countries showed that mothers who gave birth once was associated with failure to initiate breastfeeding early [19]. This can be explained by mothers having previous experience of delivery can get information about essential new born care during their previous ANC, Delivery, PNC and immunization periods and this can inspire mothers to practice essential new born care more.

Mothers who get counseling during delivery were 3.3 times more likely to practice essential new born care. Similar study conducted in East Gojjam Zone, Ethiopia found that positive association between advice about Essential New born Care practices during monthly pregnant mother’s group meeting and advice about birth preparedness during ANC visits [16]. Similarly, another study conducted in Addis Ababa showed that advice during and after pregnancy about ENC was associated with ENC practice of women [17]. This can be explained by mothers who got counseling about essential newborn care during ANC, Delivery and PNC periods can have a better understanding on the importance of essential new born care practice than their counter parts.

The result of this study implies that essential neonatal care practice is very low in the study area and more emphasis should be given by, governmental and non-governmental stakeholders as well as health professionals.

### Limitation of the study

Information was taken from mothers who gave birth six months back; therefore, it might have recall bias, and the study was not supported with qualitative design.

## Conclusion and recommendation

Around three fourth of mothers were not practicing the comprehensive Essential New born care. Occupation, parity and essential new born care counseling during delivery were factors significantly associated with utilization of essential new born care. Routine counseling to all mothers about essential new born care during ANC, Delivery and PNC, focusing on primigravida mothers and empowering women to be employed are important to promote essential new born care practice.

## Supporting information

S1 Data the minimal anonymized data set is available in the manuscript and supporting files. (DTA)
